# Insanity and Suicide

**Published:** 1874-01

**Authors:** 


### INSANITY AND SUICIDE.

INSANITY is literally the looking at a thing in unhealthful proportions. Ten years, five years, one year—a month, a day, or even a few hours later than an event, we often wonder to ourselves that we regarded it in so strong a light. How often do persons get to thinking about things at night, and become so indignant or outraged that they can scarcely remain in bed; or get to thinking about a debt they owe to-morrow in bank, and are so fearful that the money may not come in from creditors, and protest, with all its horrors, stares them in the face, and they toss and turn in an agony of sweat, and containing themselves no longer, they get up and frantically pace the floor by the hour, then falling asleep from sheer exhaustion, they wake up to see the cheerful sun shining in the windows, and the postman brings more remittances than were needed.

Many who have treated Consumption largely have died of the disease.

Hydrophobia has been brought on by the mere force of the imagination.

The celebrated Dr. Green, so famous at one time for treating diseases of the windpipe and its connections, and after swabbing the throats of others millions of times, perhaps, began at last to swab his own.

An eminent and able physician, who attended a lunatic asylum for twenty years at Lexington, Kentucky, killed himself the other day, himself a lunatic.

Several great scholars, who have made the prophecies their study, have died insane, and so with numbers who have studied perpetual motion. All men should be careful of getting into the rut of one subject, of one idea. There is at least one advantage of having a great many irons in the fire; the man who gives them equal attention never goes crazy. It is the brooding over one disagreeable thing which fills our mad houses; it is really the want of force of character, of moral courage, which leads to such a fate. The moment your sleep is disturbed about one thing, for two nights in succession, tear yourself away from it or you may be lost.

